# Risk Factors for Readmission Following Revision Total Hip Arthroplasty in Patients Undergoing Surgery for Noninfective Causes

**DOI:** 10.7759/cureus.2934

**Published:** 2018-07-06

**Authors:** Andrew S McGee, John L Johnson, Kyle D Paul, Harshadkumar A Patel, Matthew C Christie, Brooklyn D Williamson, Ashish Shah, Sameer M Naranje

**Affiliations:** 1 School of Medicine, University of Alabama at Birmimgham, Birmingham, USA; 2 School of Medicine, University of Alabama at Birmingham, Birmingham, USA; 3 Orthopaedic Surgery, University of Alabama at Birmingham, Birmingham, USA; 4 Orthopaedics, University of Alabama at Birmingham, Birmingham, USA; 5 Psychology, University of Alabama, Tuscaloosa, USA

**Keywords:** readmission, total hip arthroplasty, revision total hip arthroplasty, risk factors

## Abstract

Introduction

Readmission following revision orthopedic surgery imposes tremendous costs due to the increased length of stay, procedure complexity, and revision surgery. Following revision total hip arthroplasty, as many as one in five patients are readmitted postoperatively. Readmissions cost the federal government $17.4 billion annually. The purpose of this study was to identify risk factors for unplanned readmission following revision total hip arthroplasty.

Methods

This was a retrospective case series review of randomized revision total hip arthroplasties (THA) patients between 2008 and 2018. Exclusions were as follows: outside hospital revisions, staged revisions, revisions for infection, and bilateral revisions. Data were collected by manual chart review. Readmissions were tracked from discharge until the final follow-up.

Results

A total of 61 patients and 85 revision THAs were analyzed. Nineteen patients (31.1%) were readmitted; 31.6% of the readmitted patients had a coronary artery disease compared to 6.5% of non-readmitted patients. Readmission was also associated with obesity, former smokers, and hypertension. Also, the mean duration of follow-up was 26.5 months for readmitted patients as compared to 8.96 for non-readmitted patients.

Conclusion

Obesity, former tobacco use, younger age, coronary artery disease (CAD), and hypertension were associated with readmission. The medical optimization of patients with these risk factors prior to surgery could significantly lower costs relative to revision THA.

## Introduction

Readmission following revision orthopedic surgery imposes a tremendous economic strain on the healthcare system, primarily due to the increased length of stay, procedure complexity, and the high risk of subsequent failure [1-2]. As such, it is imperative that surgeons be cognizant of patient and surgical factors that predispose to readmission with the aim of both improving outcomes and reducing costs. Total joint arthroplasty revision provides an excellent focus for these inquiries, as previous studies centered on these procedures have demonstrated a large and increasing disease burden, significant morbidity, and immense lifetime costs to the patient and healthcare system [2-3].

Over the past two decades, the number of total joint arthroplasties has increased exponentially [4]. Despite advancements in surgical technology and postoperative care, the readmission rate following primary surgery, especially total hip arthroplasties (THAs), remains high [5]. Kurtz et al. found that between 1990 and 2002, primary THA procedures increased 50% and the rate of revisions increased by 60% [4]. In 2014, Bozic et al. revealed that THAs increased by 23% between 2005 to 2010, with a revision burden of approximately 15% [6]. Other projections estimate that the rates of revision THAs will grow to almost 100,000 per year in 2030 [7].

The economic burden of readmissions following surgical revision is especially evident when analyzing Medicare data. One in five Medicare patients is readmitted following revision THA due to complications, placing an estimated $17.4 billion strain on the United States federal government [2-3]. Furthermore, it is estimated that a 1% decrease in total joint arthroplasty revisions within the United States would translate into a cost reduction of up to $211 million per year [4]. In addition to increased cost, readmission also negatively impacts the reimbursement of physicians and hospital systems due to regulations within the Affordable Care Act (ACA). Higher rates of readmission adversely affect the quality of care index, thereby significantly reducing hospital and physician reimbursement rates [8].

Physicians need to understand the etiologies and factors putting patients at risk of readmission following revision THA to help alleviate its burden. The purpose of this study was to identify those risk factors for unplanned readmission following single-stage total hip arthroplasty revision using long-term data collected from the entirety of patient follow-up.

## Materials and methods

A retrospective case series was conducted to evaluate the rates and causes of unplanned readmissions following revision THA. A randomized selection of 150 patients who underwent revision THA procedures from 2008 to 2018 at a single academic center were identified using the Current Procedural Terminology (CPT) codes 27134 (revision total hip arthroplasty; both components, with or without autograft or allograft), 27237 (revision of total hip arthroplasty; acetabular component only, with or without autograft or allograft), 27138 (revision of total hip arthroplasty; femoral component only, with or without allograft). Data was collected by manual chart review of patients’ medical record by three authors (KP, AM, JJ). Any discrepancies encountered during record review were reconciled by a fourth author (SN). Exclusion criteria consisted of the following: patients with prior revisions at outside hospitals and staged revision procedures for infection and bilateral revisions. Staged revisions for infection were excluded to eliminate planned readmissions. After exclusions, we selected 61 patients that satisfied selection criteria. Primary tracked outcomes included surgical and medical complications, unplanned readmissions, and unplanned revision procedures. Revision was defined as a surgical procedure in which arthroplasty components were removed or exchanged or additional hardware was implanted. Simple irrigation and debridement without exchange or alteration in components were not considered revisions. Readmission was defined as presentation to the hospital emergency department with a subsequent stay of ≥ 24 hours or a direct admission from a medical provider for reasons directly related to the most recent revision. The causes for readmissions following revision were categorized as pain, hematoma at the surgical site, venous thromboembolism, instability, osteolysis, loose components, periprosthetic fracture, hardware failure, and reoperation. Demographic variables (e.g., sex, race, gender, body mass index (BMI)), medical comorbidities, preoperative diagnoses, postsurgical complications, readmitting diagnoses, and radiographic imaging were collected and included in the analysis.

## Results

**Figure 1:**
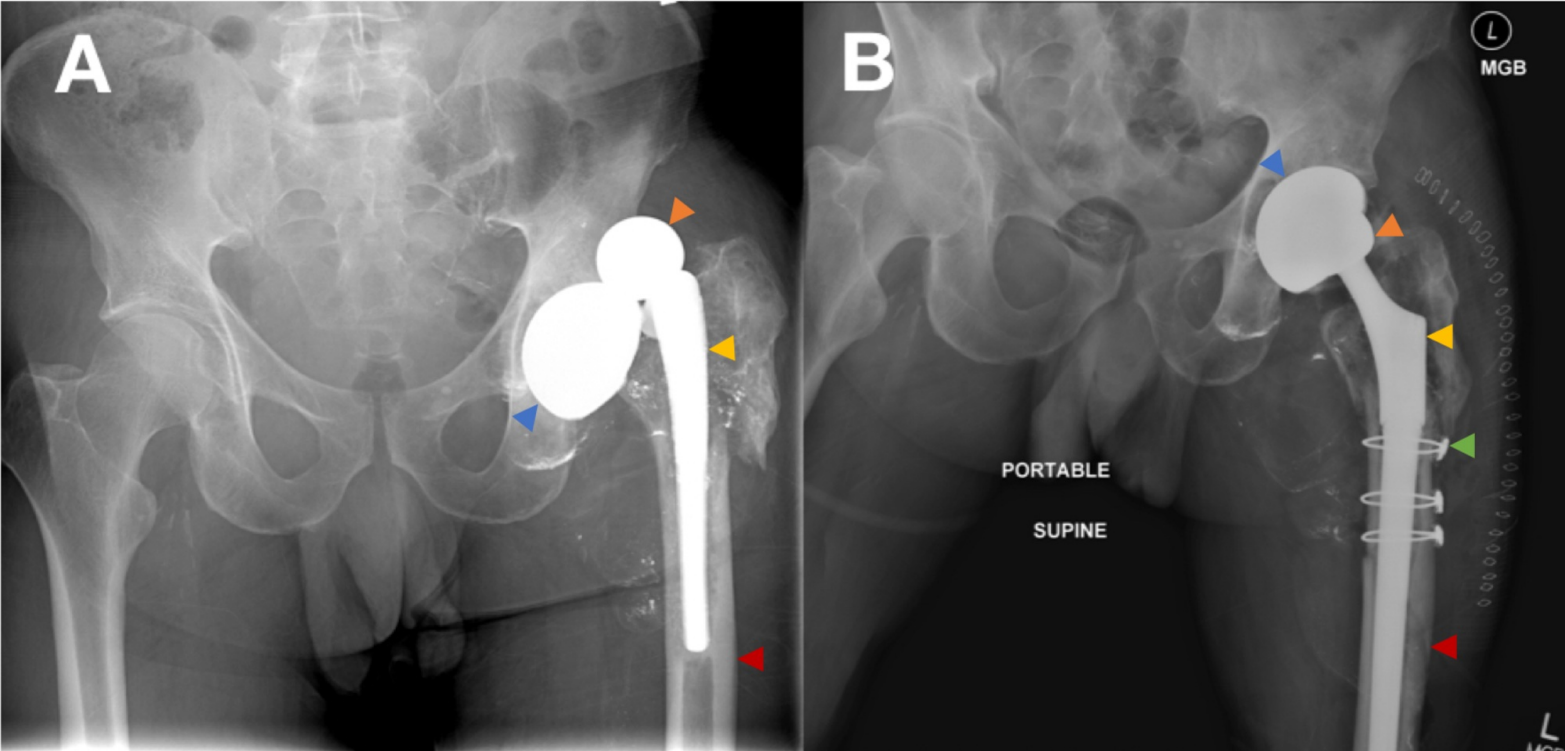
Representative Staged Radiographs for Revision Images A and B display radiographs of a patient who underwent left revision THA during the study period. Image A displays a preoperative AP pelvis radiograph prior to first revision THA. Image B displays an AP pelvis radiograph nine days status post first revision THA. The blue arrows demonstrate the acetabular cup. The orange arrows demonstrate the femoral head. The yellow arrows demonstrate the femoral component. The red arrows demonstrate the femur. The green arrow demonstrates the cerclage wires used in revision. THA: total hip arthroplasty; AP: anteroposterior

For both readmitted and non-readmitted patient groups, Table 1 shows that the most common preoperative diagnosis for revision THA was prosthesis instability (63.2% and 64.3%, p value=0.93), followed by periprosthetic fracture (15.8% and 9.5%, p value=0.477). Representative radiographic images taken preoperatively, postoperatively, and at the most recent clinic visit for a patient who underwent revision for recurrent dislocation and instability are displayed in Figure 1.

**Table 1:** Preoperative Diagnoses of First Revision THA THA: total hip arthroplasty

	Readmitted Patients (N=19)	Non-readmitted Patients (N=42)
Osteolysis	1 (5.3%)	11 (26.2%)
Instability	6 (31.6%)	6 (14.3%)
Loose Components	5 (26.3%)	10 (23.8%)
Periprosthetic Fracture	3 (15.8%)	4 (9.5%)
Symptomatic Metallosis	1 (5.3%)	3 (7.1%)
Polyethylene Wear	0	4 (9.5%)
Pain	2 (10.5%)	2 (4.8%)
Failed THA (Unspecified First Revision)	1 (5.3%)	2 (4.8%)

Of the 61 patients, 31.1% (19/61) were readmitted anytime within the follow-up period after the index procedure. The mean follow-up duration for those readmitted was 26.50 months (range, 2.79 to 87.52), as shown in Table 2. In contrast, the mean follow-up duration for those not readmitted was 8.96 months (range, 0.13 to 35.28).

**Table 2:** Duration of Clinical Follow-up After First Revision THA THA: total hip arthroplasty

Time Between First Revision and Final Clinic Visit	Mean (Months)	Standard Deviation (Months)	Median (Months)	Maximum (Months)	Minimum (Months)
Non-Readmitted Patients	8.96	8.83	4.59	35.28	0.13
Readmitted Patients	26.5	21.73	20.78	87.52	2.79

There were two patients (3.3%) readmitted within 30 days following first revision THA. For these two patients, the preoperative diagnoses were instability and pain secondary to a metal-on-metal prosthesis. The causes of their readmissions after the first revision THA were surgical site hematoma and surgical site infection, respectively.

Beyond 30 days, 31.1% (19/61) of patients were readmitted for causes related to the first revision THA. Of these patients, 84.2% (16/19) had an additional revision THA procedure after readmission. The causes for readmission that did not require further revision of implanted components include superficial surgical site infection (1), deep surgical site infection (5), and surgical site seroma (1). Causes for readmission resulting in a further revision of components include joint instability (18), hardware failure (1), periprosthetic fracture (3), and surgical site infection (2), as outlined in Table 3.

**Table 3:** Readmitted Patient Characteristics and Indications for First Revision, Readmission, and Re-revision BMI – Body Mass Index; ASA – American Society Anesthesiologists; C– Current Smoker; F – Former Smoker; N – Never Smoker; SSI – Surgical Site Infection; I&D – Irrigation & Debridement; THA – total hip arthroplasty

Patient	Age	BMI	ASA Class	Smoking Status	First Revision Preoperative Diagnosis	Reason for Readmit Within 30 Days	Reason for Readmit Beyond 30 Days	Number Re-revisions	Re-revision Preoperative Diagnoses
1	57	40.85	3	F	Instability		Superficial SSI	0	-
2	54	44.29	3	N	Periprosthetic Fracture		SSI	0	-
3	78	20.9	3	F	Instability	Surgical Site Hematoma	SSI	0	-
4	58	29.08	3	N	Osteolysis		Re-revision	1	Osteolysis & Loosening
5	63	20.3	3	F	Loose Component		Re-revision	1	Instability
6	68	29.42	3	N	Symptomatic Metallosis		Re-revision	1	Instability
7	57	31.17	3	F	Loose Component		Re-revision	1	Periprosthetic Fracture
8	51	31.32	3	N	Instability		Re-revision	1	Instability
9	69	28.07	3	F	Loose Component		Re-revision	1	Instability
10	49	29.43	3	N	Failed THA (Unspecified)		Re-revision	1	Instability
11	49	29.03	3	F	Pain	SSI	Re-revision	1	SSI
12	67	26.63	3	F	Loose Component		Re-revision	1	Periprosthetic Fracture
13	52	28.54	3	F	Pain		Re-revision	1	Acetabular Cup Failure
14	51	22.96	3	C	Periprosthetic Fracture		Re-revision (2); SSI after Third Revision	2	Instability
15	77	31.75	3	F	Instability		Re-revision (2)	2	Instability
16	62	43.55	3	N	Loose Component		Re-revision (2)	2	Instability
17	61	41.5	3	C	Periprosthetic Fracture		Re-revision; I&D Seroma; I&D Infection; Re-Revision; I&D Infection	2	Instability
18	70	31.65	3	F	Instability		Re-revision (3)	3	Periprosthetic Fracture; Instability; SSI
19	54	20.31	3	C	Instability		Re-revision (3)	3	Instability

In this cohort, there were six patients in the readmitted group with CAD and three patients in the non-readmitted group with CAD (31.6% vs. 6.5%, p = 0.021), as detailed in Table 4. Furthermore, there was a higher percentage of obese to morbidly obese former smokers and hypertensive patients in the readmitted group – though none of these factors reached statistical significance. No other correlations with readmission were identified with respect to demographic and health-related characteristics, including sex, race, American Society Anesthesiologists (ASA) class, and other preoperative comorbidities.

**Table 4:** Patient Demographic and Preoperative Characteristics ¥ Number in parentheses reports 1SD *Values not sufficient to generate P-value ASA: American Society Anesthesiologists; BMI: body mass index; COPD: chronic obstructive pulmonary disease

	Readmitted (N=19)	Non-Readmitted (N=42)	P-Value
Demographic Characteristics			
Age ¥	60.37 (9.07)	65.48 (11.41)	0.091
Sex			0.717
Male	10 (52.63%)	20 (47.62%)	*
Female	9 (47.37%)	22 (52.38%)	*
Race			0.506
White	16 (84.21%)	28 (66.67%)	*
Black	3 (15.79%)	12 (28.57%)	*
Multiple	0	1 (2.38%)	*
Other	0	1 (2.38%)	*
BMI Category			0.105
Normal (18.5 to <25 kg/m^2^)	4 (21.05%)	12 (28.57%)	*
Overweight (25 to <30 kg/m^2^)	7 (36.84%)	16 (38.09%)	*
Obese (30 to <35 kg/m^2^)	4 (21.05%)	9 (21.43%)	*
Very obese (35 to <40 kg/m^2^)	0	4 (9.52%)	*
Morbidly Obese (≥40 kg/m^2^)	4 (21.05%)	1 (2.38%)	*
Smoking Status			0.183
Current	3 (15.79%)	12 (28.57%)	*
Former	10 (52.63%)	12 (28.57%)	*
Never	6 (31.58%)	18 (42.86%)	*
Preoperative Comorbidities			
Diabetes	5 (26.32%)	11 (26.19%)	1
Hypertension (HTN)	15 (78.95%)	27 (55.10%)	0.372
Coronary Artery Disease	6 (31.58%)	3 (7.14%)	0.021
History of MI	0	1 (2.38%)	1
Heart Failure	1 (5.26%)	6 (14.29%)	0.418
COPD	2 (10.53%)	6 (14.29%)	1
Liver Disease	0	3 (7.14%)	0.546
Renal Disease	1 (5.26%)	3 (7.14%)	1
Thyroid Disease	3 (15.79%)	9 (21.43%)	0.737
Depression	6 (31.58%)	9 (21.43%)	0.522
Operative Variables			
ASA Class			0.300
2	0	4 (9.52%)	*
3	19 (100%)	38 (90.48%)	*
Length of Hospital Stay, days ¥	4.21 (2.84)	3.87 (1.82)	0.597

## Discussion

Considering the projected growth of total joint arthroplasty procedures and subsequent revision, readmission places a significant burden on patient well-being and the health care system. While prior studies utilized a 90-day readmission cutoff [9], this study reviewed each patient’s chart for risks associated with readmission over the entirety of follow-up.

In 2017, Badarudeen et al. found that unspecified mechanical complications were the most common preoperative diagnoses for revision THA within one year of the index procedure in the Medicare population (40.7%), followed by instability (14%) and infection (11.3%) [10]. Our case series found similar results with mechanical complications being the most common reasons for revision THA. Our study further subcategorized mechanical complications into instability, loose components or protrusion and malposition of components, and osteolysis. Other reasons for readmission resulting in re-revision in this study included fracture, metallosis, polyethylene wear, pain, and surgical site infection.

Mahomad et al. found a rate of readmission within 90 days following revision THA to be 10.0% in a Medicare claims database [9]. This study found the overall readmission rate to be 31.1% (19/61) with a mean follow-up of 8.96 months and 26.5 months for non-readmitted and readmitted patients, respectively. This large difference in rates of readmission may be attributable to length-time bias as well as the nature of manual chart review in comparison to large database studies. In contrast to previously published literature, this study reveals risk factors for readmission, which exist beyond the usual short-term postoperative data collection period.

Associations with readmission

A study by Wagner et al. demonstrated that BMI increased the risk of revision, infection, and dislocation within the first six months following primary THA [11]. Although this study did not identify BMI as a statistically significant independent risk factor for readmission, our exclusion of infection likely eliminates a disproportionate number of obese patients, as patients with higher BMI have been reported to have higher rates of implant revision, infections, and dislocations–all of which may lead to readmission.

This case series found that readmitted patients were younger on average than those not readmitted following revision THA. Additionally, 84.21% (16/19) of patients with readmissions required further revision surgery. This is consistent with a study by Khatod et al. that found for every 10-year increase in patient age, the hazard ratio for re-revision decreased by a factor of 0.72 [12].

This study found that a history of coronary artery disease (CAD) was a significant (p=0.021) predictor of hospital readmission following revision THA. Thus, special care should be taken for pre- and peri-operative workup for patients with previously diagnosed CAD and associated disease states (dyslipidemia, diabetes, and hypertension). Despite the increased rates of CAD within our readmitted populations, there was no significant correlation between the admitted and non-admitted populations with respect to smoking status or hypertension. The failure to reach significance is likely a consequence of the relatively small sample size as opposed to a true correlation because the relationship between coronary artery disease, smoking, and hypertension are well-established. This study further underscores the importance of aggressive medical optimization prior to elective revision surgery, especially in patients who have risk factors for readmission.

Of the patients in our study, 57 of the 61 (93.4%) had an ASA classification of 3. By definition, patients with an ASA classification of 3 have a severe systemic disease [13]. An important consideration with respect to our data is that both groups had severe systemic disease prior to surgery, as previously discussed and detailed in Table 3. Therefore, our data provide insight into which systemic diseases included in the ASA classification are associated with readmission following revision THA surgery such as CAD.

Limitations

Several limitations to this study exist. Like many retrospective case series, this study is limited by small sample size, data entry error, lack of blinding, limited randomization, and selection bias. In addition, any data regarding readmission at an outside hospital was unavailable for review.

## Conclusions

This retrospective review suggests younger age, BMI of >35, hypertension, history of tobacco use, and coronary artery disease as risk factors for readmission following revision total hip arthroplasty. This data further emphasize the importance of the medical optimization of patients with significant comorbidities prior to surgery. Future studies may focus on the role of coronary artery disease as an independent risk factor for readmission and methods for mitigating its effect.
